# Runners with patellofemoral pain demonstrate sub-groups of pelvic acceleration profiles using hierarchical cluster analysis: an exploratory cross-sectional study

**DOI:** 10.1186/s12891-018-2045-3

**Published:** 2018-04-19

**Authors:** Ricky Watari, Sean T. Osis, Angkoon Phinyomark, Reed Ferber

**Affiliations:** 1 Faculty of Kinesiology, University of Calgary, Calgary, Alberta Canada; 2 Coordination for the Improvement of Higher Education Personnel (CAPES), Brasilia, Brazil; 3 Running Injury Clinic, University of Calgary, 2500 University Drive NW, Calgary, Alberta T2N 1N4 Canada; 4 Institute of Biomedical Engineering, University of New Brunswick, Fredericton, New Brunswick Canada; 5 Faculty of Nursing, University of Calgary, Calgary, Alberta Canada

**Keywords:** Patellofemoral pain, Running kinematics, Pelvic acceleration, Gait analysis, Biomechanics, Principal component analysis, Cluster analysis

## Abstract

**Background:**

Previous studies have suggested that distinct and homogenous sub-groups of gait patterns exist among runners with patellofemoral pain (PFP), based on gait analysis. However, acquisition of 3D kinematic data using optical systems is time consuming and prone to marker placement errors. In contrast, axial segment acceleration data can represent an overall running pattern, being easy to acquire and not influenced by marker placement error. Therefore, the purpose of this study was to determine if pelvic acceleration patterns during running could be used to classify PFP patients into homogeneous sub-groups. A secondary purpose was to analyze lower limb kinematic data to investigate the practical implications of clustering these subjects based on 3D pelvic acceleration data.

**Methods:**

A hierarchical cluster analysis was used to determine sub-groups of similar running profiles among 110 PFP subjects, separately for males (*n* = 44) and females (*n* = 66), using pelvic acceleration data (reduced with principal component analysis) during treadmill running acquired with optical motion capture system. In a secondary analysis, peak joint angles were compared between clusters (*α* = 0.05) to provide clinical context and deeper understanding of variables that separated clusters.

**Results:**

The results reveal two distinct running gait sub-groups (C1 and C2) for female subjects and no sub-groups were identified for males. Two pelvic acceleration components were different between clusters (PC1 and PC5; *p* < 0.001). While females in C1 presented similar acceleration patterns to males, C2 presented greater vertical and anterior peak accelerations. All females presented higher and delayed mediolateral acceleration peaks than males. Males presented greater ankle eversion (*p* < 0.001), lower knee abduction (*p* = 0.007) and hip adduction (*p* = 0.002) than all females, and lower hip internal rotation than C1 (*p* = 0.007).

**Conclusions:**

Two distinct and homogeneous kinematic PFP sub-groups were identified for female subjects, but not for males. The results suggest that differences in running gait patterns between clusters occur mainly due to sex-related factors, but there are subtle differences among female subjects. This study shows the potential use of pelvic acceleration patterns, which can be acquired with accessible wearable technology (i.e. accelerometers).

## Background

Patellofemoral pain (PFP) is the most common musculoskeletal overuse injury in runners, regardless of sex and age [1] and it has been suggested that atypical gait kinematics may play a role in its etiology [2–4]. However, a general consensus on the pathomechanics of this injury has yet to be reached [4] possibly due to the existence of more than a single atypical gait pattern [5–8].

Distinct running kinematic sub-groups have been identified in PFP patients, with a sub-group presenting lower peak hip adduction; another with greater peak knee abduction angles [6]; and a sub-group that presented an attempt to compensate for a greater initial hip internal rotation with an external rotation during midstance [7], suggesting the existence of multiple kinematic pathomechanical pathways or motor adaptations associated with PFP. It also has been shown that kinematic differences are influenced by sex-related factors, wherein males with PFP present lower angles of hip adduction and knee abduction during running [8]. These findings should be interpreted with caution, as they are from relatively small sample sizes (*n* = 16–22) and were based on visual inspection of the data, approaches which may not fully describe the etiology of PFP and related sub-groups.

The consensus statement from the 3rd International Patellofemoral Pain Research [9] concluded that “identification of sub-groups remains the ‘holy grail’ for PFP research”. Identification of sub-groups could provide insight into the pathomechanics associated with PFP as well as inform personalized treatment. One approach to identify homogenous sub-groups within a dataset is the use of cluster analyses. With the advance of technology and data science methods the use of machine learning techniques in gait analysis is growing and exploratory analysis of complex data such as gait kinematics is important to bring new insights in the field [10, 11]. Recent research from our laboratory [12] utilized a hierarchical cluster analysis (HCA) approach to successfully identify two distinct and homogeneous kinematic sub-groups among 121 healthy runners. However, because the acquisition of 3-dimensional (3D) kinematics data is time consuming, it usually relies on multiple assessors to collect data on larger sample sizes, introducing sources of imprecision into the data collection process, especially from marker placement errors [13–15]. Therefore, finding alternative methods for evaluating gait mechanics becomes important for clinical applications.

Recently, axial segment acceleration data has provided unique insight into running mechanics, discriminating between fatigue states [16] and training levels [17]. Therefore, the analysis of pelvic acceleration profiles could also be useful to identify sub-groups of runners with PFP, with the advantage of being less influenced by identification of anatomical landmarks when using optical motion capture systems. Furthermore, the study of segmental accelerations has the option to use wearable devices for data acquisition, which are becoming increasingly popular in both academia and industry, and there has been an effort to further investigate their potential applications in health systems [18–20]. Therefore, this approach may offer an accessible and objective method of assessment with clinical applicability.

The purpose of this exploratory study was to determine if running gait patterns in PFP runners could be clustered into homogeneous sub-groups using pelvic acceleration data, using a large dataset of males and females with PFP. Based on the results from previous studies, we hypothesized that more than one running gait pattern sub-group, or cluster, would be present in female PFP runners, since the studies suggesting the existence of sub-groups were mostly comprised of women [6, 7]. Furthermore, female runners with PFP would be different from their male counterparts, given that sex-related kinematic differences have been identified previously [8]. A secondary purpose was to analyze kinematic differences between the sub-groups, by comparing lower limb peak angles that are considered important in the pathomechanics of PFP, and thereby investigate the practical and clinical implications of clustering these subjects based on 3D pelvic acceleration data. Based on the kinematic sub-groups that has been described in the literature [6, 7], we expected female clusters to present differences in hip and knee frontal and transverse plane angles, and males to display lower peak angles of hip adduction and knee abduction [8].

## Methods

### Participants

Data from 110 physically active individuals with PFP with running as their primary exercise modality for at least 6 months, were analyzed in this cross-sectional study. The presence of PFP was confirmed by a licensed healthcare professional (i.e., athletic therapist, physical therapist or medical doctor) based on specific inclusion and exclusion criteria (Table 1). Subjects experiencing pain in other sites were also included in the study, however the primary complaint had to be PFP. Data was collected either at the University of Calgary or in clinical settings partnered with the Running Injury Clinic.

**Table 1:** Inclusion and exclusion criteria^a^

Inclusion criteria	
1. Insidious onset of symptoms unrelated to trauma and persistent for at least 4 wk	
2. Pain in the anterior knee associated with at least 3 of the following:	
a. During or after activity (running and other physical activity modalities)	
b. Prolonged sitting	
c. Stair ascent or descent	
d. Squatting	
3. Pain with palpation of the patellar facets or pain during step down from a 20-cm box or during a double-legged squat	
Exclusion criteria	
1. Meniscal or other intra-articular injury	
2. Cruciate or collateral ligament laxity or tenderness	
3. Positive patellar-apprehension sign	
4. Evidence of effusion	
5. History of recurrent patellar subluxation or dislocation	
6. History of surgery to the knee joint	
7. Nonsteroidal anti-inflammatory drug or corticosteroid use within 24 hours before testing	
8. History of head injury or vestibular disorder within the last 6 months	
9. Pregnancy	

^a^adapted from Ferber et al. (2015) [3]

### Data collection

The data collection methods are described in detail elsewhere [21, 22]. Briefly, 8 high-speed digital video cameras (MX3/Nexus, Vicon, Oxford, UK) were used to film treadmill-running at 200 Hz. Spherical retro-reflective markers (9 mm diameter, Mocap Solutions, Huntington Beach, USA) were attached to the specific lower extremity anatomical landmarks bilaterally along with technical marker clusters on rigid shells placed to represent the pelvis and bilateral foot, shank, and thigh segments. Each participant wore the same shoes (Pegasus, Nike, Beaverton, USA) to standardize the footwear condition.

Following placement of all the anatomical and segment markers, each participant stood on a motorized treadmill (Bertec Corporation, Columbus, OH, USA) for a 1-s static trial. Upon completion of the static trial, the markers on the anatomical landmarks were removed while the technical marker clusters remained. The participants were instructed to warm-up on the treadmill for 2–3 min, and then ran on the treadmill at a comfortable self-selected pace (2.61 ± 0.20 m/s) for 20 s, in which approximately 60–80 consecutive running steps were collected for processing and analysis. All participants were experienced treadmill users and were permitted as much time as they required to familiarize themselves with treadmill running before beginning the data collection.

### Data processing

Ankle, knee and hip joint sagittal plane angular accelerations were used for defining ground contact, using previously published event detection methods [23]. The position of the pelvis was measured using the centroid of the pelvic marker cluster [24] and pelvic acceleration was calculated by double differentiation of pelvis displacement using a modified Savitzky-Golay method [25]. Differentiation was performed at both stages using a time-window of 10 data points, and 4th order polynomial fitting. In order to emulate a wearable device, marker accelerations in the global coordinate frame were then converted to a local coordinate frame on the pelvis, using segment markers and rigid body transformations [26]. The local coordinate frame was aligned with the global frame during the static trial.

Each step cycle was normalized to 100 points, with 80 data points for stance and 20 data points for flight phase, since we are analyzing an axial segment. These normalized phases were then combined to represent 100% of the step cycle, averaged over all extracted steps, and standardized to zero mean and unit variance. The kinematic data (3 planes of motions × 100 time-normalized pelvic accelerations) were combined into one 300-dimensional row vector for each subject, creating a matrix of 110 subjects-by-300 data points.

### Data analysis

The HCA method was used to identify homogeneous running gait patterns separately for males and females based on the pelvic acceleration time-series, by creating a cluster tree, or dendrogram for each sex-group. Agglomerative strategy or a “bottom up” approach was used, which consists of three steps: (1) a measure of dissimilarity between sets of subjects using the Euclidean distance, (2) subject linkage using the Ward’s minimum variance method [27], and (3) cluster determination using the variance ratio criterion [28].

Following identification of homogeneous clusters (sub-groups) of PFP runners, differences in demographics, injury characteristics, vertical displacement of the pelvic centroid and peak joint angles were examined using one-way ANOVA (Tukey test for post-hoc analyses) and chi-squared test (*α* = 0.05), and effect sizes were calculated based on *η*^*2*^ and Cramer’s *V* indices, respectively. In case the data did not present a normal distribution (Shapiro-Wilk test) or a homogeneous variance between sub-groups (Levene test), the Kruskal-Wallis test was performed (Dunn’s test for post-hoc analyses). Differences in pelvic acceleration patterns were examined after applying a principal component analysis (PCA) to the standardized data matrix, and they were identified based on the interpretation of principal components (PCs) that presented a large effect size (*η*^*2*^ > 0.14) [12, 29], which were used to reconstruct the acceleration waveforms for a better mechanical interpretation [30]. The squared coefficients of correlations between the PC scores and the raw acceleration data (squared loading) [31] were used to calculate the relative loading of the PCs in the vertical (VT), antero-posterior (AP) and medio-lateral (ML) directions to aid in the interpretation of the PCs [32].

We also selected joint angles that are considered important in PFP pathomechanics and that have been suggested to differ between PFP sub-groups [6, 33], to compare between sub-groups. The analyzed peak joint angles were: ankle eversion; knee flexion, knee abduction and knee external rotation; and hip adduction and internal rotation.

A Pearson’s correlation coefficient was calculated between the significant PCs and demographic, injury characterization and kinematic variables that presented differences between sub-groups to determine whether these latter factors were significantly correlated with the acceleration patterns. All data processing and statistical analysis were performed on MATLAB 9.1 (The MathWorks Inc., Natick, MA,USA).

## Results

### Identification of PFP sub-groups

**Fig. 1:**
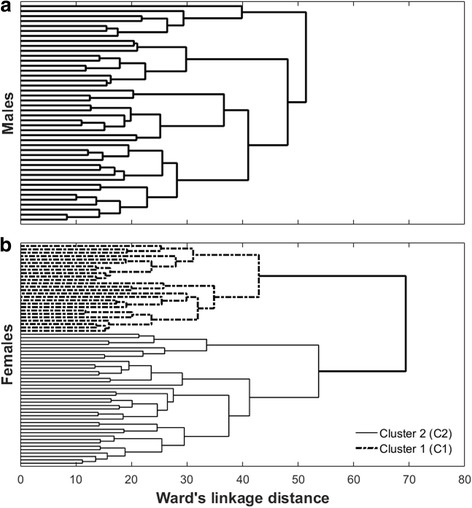
Dendrogram of the hierarchical cluster analysis. Clustering of PFP patients produced by the hierarchical cluster analysis. **a** Male subjects; (**b**) Female subjects

For the female subjects, the variance ratio criterion determined the optimal number of clusters to be two sub-groups (C1 and C2) (Fig. 1a), whereas for the male subjects, no sub-groups could be identified (Fig. 1b) in the HCA.

Subject clinical and demographic characteristics for each cluster are presented in Table 2. There was a significant difference in height (*H* = 49.9; df = 2; *p* < 0.001; *η*^*2*^ = 0.32), with male subjects being taller than both female clusters (*p* < 0.001 for males vs C1, and males vs C2); and also in mass (*H* = 50.2; df = 2; *p* < 0.001; *η*^*2*^ = 0.32), with male subjects being heavier than both female clusters (*p* < 0.001 for males vs C1, and males vs C2). There were no significant differences between clusters with respect to age (*F* = 2.4; df = 2; *p* = 0.095; *η*^*2*^ = 0.04), running speed (*H* = 1.3; df = 2; *p* = 0.521; *η*^*2*^ = 0.01), years running (*H* = 0.9; df = 2; *p* = 0.629; *η*^*2*^ = 0.01); ratio of unilateral to bilateral involvement (χ^2^ = 1.7; *p* = 0.434; *V* = 0.12) or subjects with multiple injury sites (χ^2^ = 0.5; *p* = 0.781; *V* = 0.07).

**Table 2:** Number of PFP participants and subject specifications (Mean and (SD)) for the determined clusters

	Males (*n* = 44)	Females_C1 (*n* = 26)	Females_C2 (*n* = 40)
Age [years]^a^	35.1 (1.5)	30.9 (2.0)	36.4 (1.6)
Height [m]^b^	1.79 (0.01)*	1.66 (0.01)	1.66 (0.01)
Mass [kg]^b^	77.2 (1.3)*	59.1 (1.7)	63.4 (1.4)
Running speed [m/s]^b^	2.66 (0.03)	2.60 (0.04)	2.57 (0.03)
Years running [years]^b^	8.6 (8.0)	7.0 (7.1)	9.0 (7.7)
Involvement [uni/bilateral]^c^	20 / 24	13 / 13	14 / 26
Injury site [single/multiple]^c^	33 / 11	19 / 7	32 / 8

^a^One-way ANOVA; ^b^ Kruskal-Wallis test; ^c^ chi-squared test; * significantly different from other 2 groups

Table 3 presents descriptive statistics for each cluster regarding pelvic acceleration components, vertical displacement and lower limb kinematics. There were significant differences with large effect sizes between sub-groups only for the following principal components of pelvic acceleration: PC1 (*F* = 39.7; df = 2; *p* < 0.001; *η*^*2*^ = 0.43), with the distinct group being the females in C2 (*p* < 0.001 for males vs C2, and C1 vs C2); and PC5 (*F* = 19.8; df = 2; *p* < 0.001; *η*^*2*^ = 0.27), wherein C1 was the sub-group with significant difference (*p* < 0.001 for males vs C1, and C2 vs C1). However, these PCs explained less than 25% of the variance in the dataset (17.0% and 7.7%, respectively). Height presented a significant correlation with PC1 (*p* = 0.016), but not with PC5 (*p* = 0.064), and the correlation coefficients were weak (*r* < 0.30). Although, body mass was different between subgroups, it had no significant correlation (*p* > 0.05) with either of the selected PCs.

**Table 3:** Mean and standard deviation of PC scores, vertical displacement and peak joint angles

	Males (*n* = 44)	Female C1 (*n* = 26)	Female C2 (*n* = 40)
PC 1 [a.u.] ^a^	2.84 (0.8)	4.6 (1.1)	−6.1 (0.9)*
PC 5 [a.u.] ^a^	1.4 (0.6)	−4.5 (0.8)*	1.4 (0.7)
Vertical displacement [mm] ^a^	104.7 (2.1)	88.8 (2.7)*	98.9 (2.2)
Ankle eversion [^o^] ^b^	7.2 (0.6)*	4.1 (0.8)	4.2 (0.7)
Knee flexion [^o^] ^b^	44.6 (0.9)	43.2 (1.2)	44.2 (0.9)
Knee abduction [^o^] ^b^	**9.3 (0.7)** ^*^	11.9 (0.9)	12.4 (0.7)
Knee external rotation [^o^] ^a^	10.0 (1.4)	11.1 (1.8)	7.6 (1.4)
Hip adduction [^o^] ^a^	8.2 (0.7)*	10.9 (0.9)	11.6 (0.7)
Hip internal rotation [^o^] ^a^	**12.7 (1.1)** ^**#**^	**18.3 (1.4)** ^**#**^	15.8 (1.1)

^a^One-way ANOVA; ^b^ Kruskal-Wallis test; * significantly different from the other 2 groups; ^#^ significant difference between the indicated groups

Bold number indicates a large effect size (*d* > 0.8)

### Differences in running kinematics between sub-groups

There was a significant difference in peak ankle eversion (*H* = 15.1; df = 2; *p* < 0.001; *η*^*2*^ = 0.12), wherein male PFP subjects presented greater angles than C1 (*p* = 0.003) and C2 (*p* = 0.004), and this joint angle had a low but significant correlation with PC1 (*p* < 0.036, *r* = − 0.20), but not with PC5. Peak knee abduction was also significantly different between clusters (*H* = 12.3; df = 2; *p* = 0.002; *η*^*2*^ = 0.09), with males exhibiting lower angles when compared to C1 (*p* = 0.019) and C2 (*p* = 0.005), and this joint angle was also only correlated with PC1 (*p* < 0.001, *r* = 0.38). There were also differences in hip adduction (*F* = 6.5; df = 2; *p* = 0.002; *η*^*2*^ = 0.11), with lower angles for male subjects in comparison to C1 (*p* = 0.043) and C2 (*p* = 0.003). The same tendency occurred for hip internal rotation (*F* = 5.2; df = 2; *p* = 0.007; *η*^*2*^ = 0.09), however the difference was only significant for males compared to women in C1 (*p* = 0.006). While peak hip adduction was correlated with PC1 (*p* = 0.005, *r* = − 0.27), hip internal rotation demonstrated a correlation with PC 5 (*p* = 0.014, *r* = − 0.23). There were no significant differences for knee flexion (*H* = 2.2; df = 2; *p* = 0.331; *η*^*2*^ < 0.01) and external rotation (*F* = 1.4; df = 2; *p* = 0.251; *η*^*2*^ = 0.03). Vertical displacement of the pelvis presented differences between sub-groups (*F* = 11.1; df = 2; *p* < 0.001; *η*^*2*^ = 0.17), wherein females from C1 displayed lower magnitudes of displacement when compared to males (*p* < 0.001) and females in C2 (*p* = 0.011); and this variable was only correlated with PC5 (*p* = 0.026, *r* = 0.21).

**Fig. 2:**
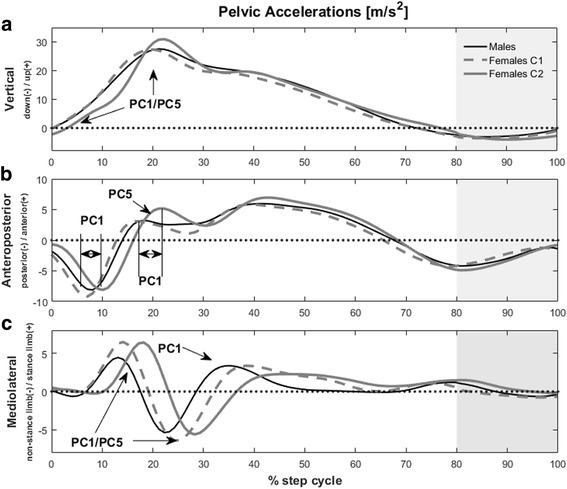
Time-normalized pelvic accelerations. **a** Vertical acceleration, (**b**) Anteroposterior acceleration, and (**c**) Mediolateral acceleration for males and female sub-groups C1 and C2 during stance phase (1%–80%) and flight phase (81%–100%; gray area) of running. Regions represented by the significant principal components are indicated in the graphs

PC1 presented a high relative loading in the VT direction (47.9%), representing variations in the peak acceleration and the magnitude at early stance phase (Fig. 2a). There was a lower relative loading of PC1 in the AP direction (28.9%), wherein it represented a phase shift of the posterior acceleration peak in early stance (Fig. 2b). In the ML direction, PC1 also represented phase shifts in ML peak accelerations towards the stance and swing limbs during the first half of stance phase (Fig. 2c), but it was the lowest relative loading (23.2%).

PC5 also had relatively high loadings in the VT axis (43.0%), denoting a difference in the rate of magnitude decrease after the peak acceleration (Fig. 2a), although these differences are subtle. The AP relative loading was the lowest for PC5 (25.0%) and it indicated a magnitude difference in the forward acceleration after weight acceptance (Fig. 2b). In the ML direction, there was a low relative loading (32.1%), representing a difference in magnitude variation during the first half of stance phase (Fig. 2c).

Overall, when comparing the pelvic acceleration patterns, males had similar acceleration patterns to females in C1 in the VT and AP directions, but the latter presented higher and delayed peaks ML accelerations. Females in C2 displayed lower acceleration magnitudes in early stance and a higher peak acceleration in the VT direction; a greater forward peak in early stance; and delayed peak accelerations in the AP and ML directions.

## Discussion

### PFP sub-groups based on pelvic acceleration

The first purpose of the present study was to determine if running gait patterns in individuals experiencing PFP at the time of testing could be clustered into homogeneous sub-groups based on combinations of pelvic acceleration components. In support of our hypothesis, two distinct and homogenous sub-groups (clusters) were present in females with PFP, and these clusters were different when compared to PFP males. These results are similar to previous studies that also reported two to three different running patterns based on visual inspection of 3D kinematic data [6, 7] and mechanical differences between males and females with PFP [8].

There were no significant differences in running speed between sub-groups, which is a factor that has been shown to affect axial segment acceleration [34], especially in the ML axis [35]. Male subjects were significantly taller and heavier than females and these anthropometric differences are known to influence 3D kinematics during running [36]. However, there was a very weak correlation for height, and no correlation for body mass with the acceleration PCs that presented differences between sub-groups suggesting that the relationship with those factors was minimal.

The advantage of investigating pelvic acceleration as a measure of running mechanics is that it is less influenced by marker placement errors and is a much simpler method than a full 3D gait assessment, as it depends only on the trajectory of a single pelvic marker cluster. Additionally, these factors allow for the use of data from multiple research centres, allowing for the application of ‘big data’ analytics and a better understanding of the interaction between biomechanical factors and musculoskeletal injuries [10, 11]. Furthermore, the results of the present study opens the possibility for the use of wearable devices for data acquisition, such as a single triaxial accelerometer on the pelvis, an approach which is becoming increasingly popular in industry and health care [18, 20]. Therefore, the current work identifying sub-groups of PFP patients is a novel finding that can guide future studies in providing better context that can hopefully improve clinical practice.

### Identification of differences in running gait patterns between sub-groups

A secondary purpose was to analyze peak joint angles between clusters to better understand the practical and clinical implications of clustering subjects with PFP based on 3D pelvic acceleration data. In general, differences in joint kinematics were sex-related, since there were no significant differences between female clusters, except for peak hip internal rotation. Moreover, the magnitude of mean differences were within the threshold for detectable kinematic changes reported by Osis et al. [15] for knee abduction (3.4^o^) and hip internal rotation (5.6^o^). However, the differences in ankle eversion and hip adduction between males and females are greater than the error margins caused by marker placement errors, confirming the findings of Willy et al. [8] who reported males with PFP to have less hip adduction than their female counterparts.

Phinyomark et al. [12] reported the existence of two different sub-groups of asymptomatic runners based on a HCA of lower limb joint kinematics, and when they compared the peak knee abduction angles of those clusters with a sample of subjects with PFP, group differences were dependent on the cluster of healthy individuals that was used as reference. Interestingly, all PFP sub-groups from the current study presented greater values of knee abduction when compared to the ones reported for healthy runners (healthy C1: 8.0^o^; healthy C2: 4.4^o^). However, there is a tendency for a progressively greater alteration in knee frontal plane angles when comparing males to females in C1 and C2, although there was no significant difference between the female clusters. This could be related with distinct pathomechanical pathways or differences in response to treatment. For example, in a previous work [37] we found that non-responders to exercise treatment protocol presented greater knee abduction angles during late stance and swing phases of running gait, and the current findings suggest that this could be identified by pelvic acceleration data.

To our knowledge, this is the first study to investigate pelvic acceleration profiles in runners with PFP, and the identification of sub-groups could generate insights about differences in pathomechanics or adaptations to pain. Additionally, the analysis of segmental acceleration profiles minimizes measurement imprecisions originating from marker placement errors that propagate into the calculation of joint angles in 3D kinematics [14, 15]. Furthermore, the results of the current study suggest that accelerations acquired using wearable devices [24] may utilise this method in a clinical setting as an evidence-informed method to improve patient care and rehabilitation decisions.

The pelvic acceleration data can provide some clinical insight that can help clinicians make decisions regarding treatment options. For example, peak resultant pelvic acceleration is related to center of mass acceleration during 10 to 75% of stance phase [38]. Therefore, pelvic accelerations can provide some insights on shock absorption and lower limb stiffness. Nevertheless, this connection must be made with caution, since accelerations based on segmental measures overestimate the behavior of center of mass [38]. Women in C2 presented a higher VT peak acceleration, suggesting a diminished capacity for shock absorption. Since no differences in peak knee flexion angles were detected, this could be an indication of greater leg stiffness in these subjects, which is partially supported by the findings that women present higher leg stiffness during running [39] and drop jump landing tasks [40] when compared to males. In contrast, females in C1 were similar to males regarding VT acceleration patterns, which could be explained by the lower VT displacement.

Women also presented higher and delayed peak accelerations in the ML direction, suggesting differences in the control of side-to-side body movement during the first half of the stance phase, when these oscillations occur. This pattern could be related to the larger hip adduction angles exhibited during running, which led to increases in ML accelerations. In addition, females in C2 displayed a delay in peak AP accelerations in early stance, causing a prolonged period of deceleration. It is possible that this finding is related to strength differences between males and females [41, 42], as stronger individuals may be able to exert shorter impulses to achieve the same net change in momentum, however, strength differences were not quantified in the current study.

Although the identification of sub-groups among the female subjects with PFP did not coincide with significant differences in peak lower limb joint angles, there seems to be a progression of values in knee abduction and hip internal rotation depending on the cluster of female subjects. Specifically, there is a tendency for C1 to have lower knee abduction and higher hip internal rotation than C2. These factors could be related to symptom severity or differences in response to treatment, but would need further investigation.

### Limitations

In addition to the differences in height and weight between males and females that were already discussed, other limitations to the current research study are acknowledged. First, this study included both subjects with uni- or bilateral involvement and with secondary pain symptoms besides PFP, which could have also modified running mechanics. However, there was no significant difference in the distribution of those variables between the two subgroups, leading us to believe that it was not an important factor for this clustering. Additionally, these types of patients are frequently seen in clinical practice, therefore these PFP patients are important to include in research studies.

Second, we did not have access to other clinical variables that could influence running mechanics and explain the differences that were found between sub-groups. For example, Selfe et al. [43] has described 3 clusters of PFP patients that were grouped based on clinical measures of strength, flexibility and joint alignment and mobility. Additionally, experimental pain induction in the knee joint has been shown to cause reductions in peak torque in maximal voluntary contraction of knee flexors and extensors [44] and increased sway displacement during quiet stance [45], indicating that pain level could be a driver of changes in motor control. Therefore, future studies should include the aforementioned clinical variables to investigate whether they are related to the differences in running pattern found between sub-groups to have a better understanding in a clinical context.

Finally, this investigation used an HCA approach, which is an unsupervised machine learning technique suitable for exploratory analyses, to determine whether this type of data could be useful in the identification of subgroups within a cohort of runners with PFP. Overall, our hypothesis was supported by the findings and suggest that a supervised analysis could also be applied to identify specific subgroups with specific clinical relevance. For example, recent work from our laboratory used a supervised machine learning method to classify runners with PFP into responders or non-responders to exercise treatment based on running kinematic data, achieving 78% of classification accuracy [37]. Thus, a similar approach could be applied in this context, using pelvic acceleration data to develop an objective method for the identification of such subgroups with greater accessibility in a clinical setting. Regardless, the present study is an important first step to verify the utility of simple measures, like pelvic accelerations, for the objective assessment of gait biomechanics.

## Conclusions

In conclusion, using a hierarchical cluster analysis, the present study is the first to identify distinct pelvic acceleration patterns during running gait in a large group of PFP runners. Two homogenous female sub-groups were identified based on pelvic accelerations with one sub-group demonstrating a delay in the posterior and mediolateral acceleration peaks compared to the other. However, both female sub-groups presented greater acceleration peaks than males in all directions. Further analysis of peak kinematic angles provided clinical context to these sub-groups and revealed that gender-differences hip internal rotation, an important factor related to PFP, is distinct among the female sub-group. These results suggest that the variability observed in running gait patterns for PFP runners occur mainly due to sex-related factors, but there are subtle differences among females that could influence the interpretation of kinematic data. The findings also highlight potential for the use of data acquired with accessible wearable technology in the identification of sub-groups in PFP patients. Future research can use this approach in order to classify PFP patients and develop targeted intervention and injury prevention strategies.

## Abbreviations

3D: Three-dimensional
AP: Anteroposterior
HCA: Hierarchical cluster analysis
ML: Mediolateral
PCA: Principal component analysis
PCs: Principal components
PFP: Patellofemoral pain
VT: vertical
